# The Role of Natural Compounds in Optimizing Contemporary Dental Treatment—Current Status and Future Trends

**DOI:** 10.3390/jfb14050273

**Published:** 2023-05-14

**Authors:** Dana Gabriela Budala, Maria-Alexandra Martu, George-Alexandru Maftei, Diana Antonela Diaconu-Popa, Vlad Danila, Ionut Luchian

**Affiliations:** 1 Department of Implantology, Removable Prostheses, Dental Prostheses Technology, Faculty of Dental Medicine, “Grigore T. Popa” University of Medicine and Pharmacy, 700115 Iași, Romania; dana-gabriela.bosinceanu@umfiasi.ro (D.G.B.); antonela.diaconu@umfiasi.ro (D.A.D.-P.); 2 Department of Periodontology, Faculty of Dental Medicine, “Grigore T. Popa” University of Medicine and Pharmacy, 16 Universității Street, 700115 Iași, Romania; ionut.luchian@umfiasi.ro; 3 Department of Dento-Alveolar Surgery and Oral Pathology, “Grigore T. Popa” University of Medicine and Pharmacy Iași, Universitatii Street 16, 700115 Iași, Romania; vlad.danila@umfiasi.ro

**Keywords:** natural products, herbal dental therapy, alternative medicine

## Abstract

For a long period of time, natural remedies were the only ailment available for a multitude of diseases, and they have proven effective even after the emergence of modern medicine. Due to their extremely high prevalence, oral and dental disorders and anomalies are recognized as major public health concerns. Herbal medicine is the practice of using plants with therapeutic characteristics for the purpose of disease prevention and treatment. Herbal agents have made a significant entry into oral care products in recent years, complementing traditional treatment procedures due to their intriguing physicochemical and therapeutic properties. There has been a resurgence of interest in natural products because of recent updates, technological advancements, and unmet expectations from current strategies. Approximately eighty percent of the world’s population uses natural remedies, especially in poorer nations. When conventional treatments have failed, it may make sense to use natural drugs for the treatment of pathologic oral dental disorders, as they are readily available, inexpensive, and have few negative effects. The purpose of this article is to provide a comprehensive analysis of the benefits and applications of natural biomaterials in dentistry, to gather relevant information from the medical literature with an eye toward its practical applicability, and make suggestions for the directions for future study.

## 1. Introduction

### 1.1. History

As they affect so many people, oral and dental disorders and anomalies are recognized as major public health concerns [1]. In 1946, Alexander Fleming issued the following warning: “There is probably no chemotherapeutic agent to which, in adequate circumstances, the bacteria cannot react by, in some manner, gaining resistance”.

Herbal therapy has been used to treat oral health issues for thousands of years, with the oldest accounts coming from India and China. It has been said that Hippocrates recommended rinsing your mouth with a mixture of alum, salt, and vinegar. Talmud, the ancient religious text, progressing back over 1800 years, suggests using a combination of “dough water” and olive oil to maintain good mouth hygiene. Pedanius Dioscorides, a Greek physician, suggested a mixture of wine, milk, and a herbal extract made from olive tree leaves and pomegranate [2]. The Arak tree, from which comes “Miswak”, has been used for dental hygiene for millennia and is still being utilized today in many Asian and African civilizations [3].

Natural substances, in addition to more traditional treatments, have been widely included in oral care products in recent years. Both the physicochemical and medicinal properties of these molecules are intriguing. These days, several companies use natural substances in their products to boost their medicinal value.

The majority of bioactive chemicals used for medicine and therapy have traditionally come from the natural world. Over the past few years, the dental care literature has paid increasing attention to natural therapeutic products alongside commercially created alternatives [4]. Because of their accessibility, safety, low cost, biocompatibility, and wide range of biological activities, natural compounds such as these are increasingly being favored over their synthetic counterparts.

### 1.2. Recent Data

Current advancements in stem cell treatment and other forms of cutting-edge technology in the field of natural medicine have garnered increased attention [5]. Natural biomaterials have been used in primary areas of regenerative medicine. Herbal agents have made a significant entry into oral care products in recent years, complementing traditional treatment procedures. The physicochemical and therapeutic properties of these substances are intriguing. Several companies now use botanical components in their wares for supplementary medicinal benefits [6].

**Figure 1:**
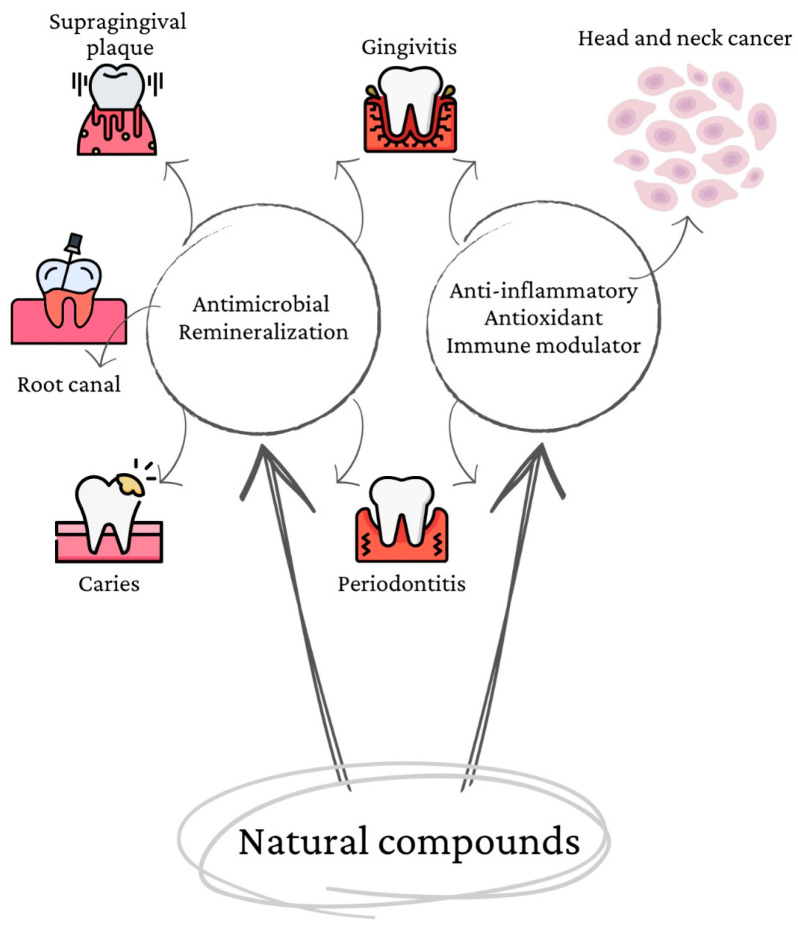
Natural compounds and their properties.

In cell cultures and preclinical animal models, numerous natural compounds have been shown to have chemo-preventive action [7,8,9]. These promising in vitro and in vivo effects cannot be replicated in clinical settings due to inadequate systematic administration and bioavailability [10]. Figure 1 below exhibits the main mechanisms of action:

Over 80% of people throughout the world utilize herbal medicines for health purposes today, with the bulk of these people living in rural areas of poor nations [11]. The use of these resources and procedures is not without its drawbacks and challenges. Most of these materials fall short of ideal performance in one or more crucial areas. There are advantages and disadvantages to all of them. Among the many difficulties faced by scientists in this sector are the limitations of natural compounds in biocompatibility, the tendency of microbial contamination, and the absence of appropriate safety data.

The goals of this review article were to provide a thorough assessment of the effects and uses of natural biomaterials in dentistry, to extract important data from the medical literature from a perspective of practical applicability, and to provide recommendations for future research.

## 2. Mechanisms of Action

Herbal treatments are complex systems in and of themselves, demonstrating a fixed network of interactions with other complex systems (the live organism). Modulating self-organization and emergence in biological systems is possible due to the fact that the changes induced by herbal combinations involve many mechanisms that promote multi-stability and produce bidirectional feedback across distinct levels of the organization. This may help to explain why seemingly little input can have far-reaching consequences and why seemingly catastrophic perturbations can often cause just a ripple impact across the system.

Herbal therapy is not a placebo and often uses active natural compounds (so-called secondary metabolites (SMs)) that are present in all plants and have a wide range of molecular weights and structural variety to treat infections and health issues. Identifying and characterizing a wide variety of SMs and their individual and synergistic mechanisms of action present a significant task for modern pharmacology. Investigating the phytochemistry of plants utilized in traditional medicine systems across the world and providing an explanation of whether or not their SMs may contribute to observed pharmacological activity is another intriguing topic.

### 2.1. Physical Disruption of Cell Membranes

A number of naturally occurring substances have been shown to disrupt membrane function and/or integrity via physical processes. Finding antibiotics that can counteract resistance phenomena might greatly benefit this method. Combining antibiotics with adjuvants or antimicrobials picked from the reservoir of natural bioactive chemicals is an attractive developing approach that has the potential to increase antibiotic effectiveness and/or eliminate antibiotic resistance by working on the defenses of a cell.

### 2.2. Covalent Modification of Proteins and DNA Bases

Many SMs possesses particularly reactive functional groups in their structures which can form covalent interactions with proteins, peptides, and sometimes DNA. Enzymes, receptors, transcription factors, ion channels, transporters, and cytoskeletal proteins are all examples of proteins that serve as cellular targets. Receptors and enzymes lose their ability to bind to their ligand or substrate if this part of their structure is altered. Because of this, SMs with reactive functional groups can target several proteins in an organism without being selective.

### 2.3. Non-Covalent Modification of Proteins

Most herbal remedies include phenols and polyphenols, which are responsible for modulating proteins: the primary target in cells. In the same way as SMs with reactive functional groups can produce covalent bonds that may influence proteins as multitarget medicines, so can SMs form multiple hydrogen and ionic bonds.

### 2.4. Surface Tension Modifiers

Several cellular and intracellular organelle membrane-associated biochemical activities are influenced by surface tension [12]. Surfactant proteins and molecules are abundant in biological fluids and on the surfaces of cells and their membranes.

With the exception of endodontics, where lowering the surface tension increases the strong interaction of irrigating solutions with the tooth structure, allowing for deeper penetration, surface tension modifiers have received little to no attention up to this point.

### 2.5. Miscellanea

In many additional cases involving both local and systemic applications, such as the local application of foams and creams, natural molecules have been demonstrated to be advantageous by exerting important therapeutic effects via physical processes.

**Figure 2:**
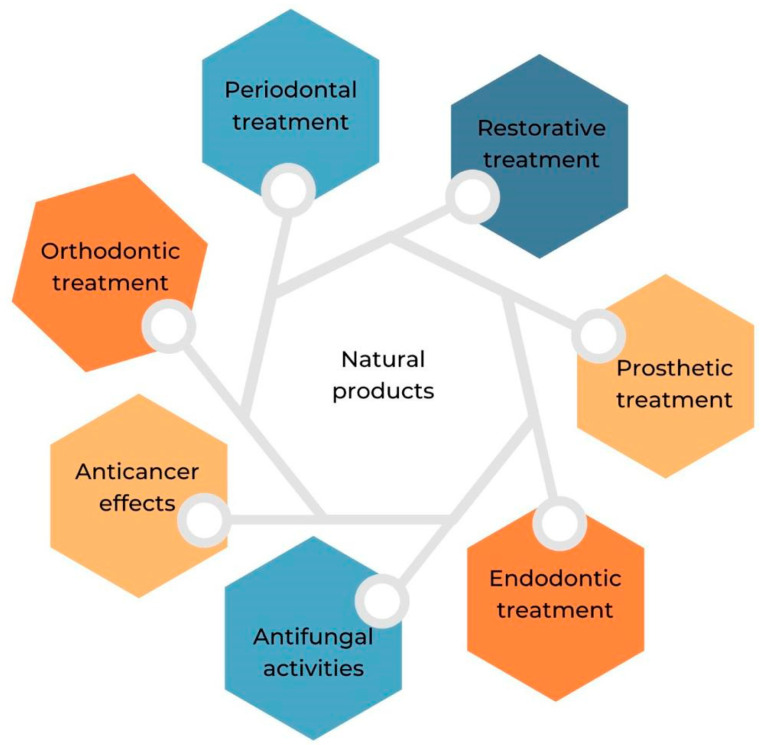
Natural compounds and their applications in dentistry.

As can be shown in Figure 2, natural products have tremendous potential as adjuvant therapy for the prevention and control of dental problems. However, numerous and major obstacles need to be addressed before the promise of natural products can be translated into clinically viable oral care solutions for the treatment of dental disorders.

## 3. Herbal Abilities to Inhibit Cariogenic Activities

For thousands of years, people all around the world have turned to herbs as a natural remedy for disorders such as toothache pain. Because of this, the quest for effective solutions continues, and phytochemicals isolated from herbs show promise as a viable alternative source [12].

Microbes have an impact on every aspect of human existence. The oral cavity is home to a wide variety of microorganisms that work together for the benefit of the host by discouraging the growth of pathogens [13]. Dental caries, periodontal disease, and pulp pathosis are only a few of the oral problems that have been associated with microbial flora imbalance [14] and oral cancer [14]. High numbers of cariogenic bacteria are one of the main etiological elements for the onset and development of dental caries, despite the fact that this disease has other causes. *Streptococcus mutans* is the most commonly found bacteria in dental plaque samples taken from humans. Its aciduricity, acidogenicity, and adhesion capability [15] are all examples of virulence characteristics that contribute to the pathogen’s invasiveness.

*Enterococcus faecalis* is another oral pathogenic bacterium that has been associated with persistent apical periodontitis in cases of unsuccessful endodontic therapy [16]. *Staphylococcus aureus* is another bacterium of dental significance since it may colonize the mouth in both healthy and diseased states [17].

In light of rising antibiotic and chemotherapeutic drug resistance, it is critical that dental disease be prevented and treated with solutions that are both reliable and effective. Herbal, animal, and microbial natural products all show promise in this regard [18]. Herbal medicine’s sudden rise in popularity can be attributed to the benefits it offers and the fact that it does no harm to the user [19,20].

Tannins, phenolic compounds, saponins, minerals, antioxidants, flavonoids, vitamins, and macronutrients are only some of the many beneficial components that have been found in herbal plants [21]. These natural chemicals have been shown to have a wide variety of mechanisms of action, including the suppression of ATP synthesis or energy metabolism, the acceleration of enamel remineralization, the prevention of colony formation and colony adhesion, and the modification of pH homeostasis [20,21].

One investigation suggested that the antibacterial activity of essential oil in sage and lemongrass may contribute to the inhibition of bacterial colonization induced by the death of its planktonic cells. These bioactive components may cause leaks in bacterial cell walls by diffusing through the peptidoglycan membrane. Thus, the death of bacterial cells occurs because they lose essential components and atoms from the plasma [22,23].

Fernandes et al. [24] reported similar results, showing that guava leaf extract might impede the development of *Escherichia coli* and *S. aureus*. Thus, it inhibits the growth of both Gram-negative and Gram-positive bacteria. The main compounds identified in guava leaf extract were phytol, acetate, ursodeoxycholic acid, neophytadiene, ursodeoxycholic acid, leden, and a-cedrene and so toothpaste, mouthwash, and dental gel can all benefit from this composition due to its antibacterial properties, which can then be used to treat a variety of oral ailments [25]. In clinical settings, mouthwashes made from tea have been shown to have remarkable antimicrobial activity in the treatment of caries. A total of forty young toddlers were tested in clinical research using herbal mouthwash versus a placebo. Salivary *S. mutans* count decreased significantly after 2 and 4 weeks of twice-daily 8 mL/day Camellia Sinensis mouthwash treatment [26]. C. Sinensis and Propolis extract had comparable effects in another investigation involving children. It has been shown that both green and black tea, which use the same plant, is effective against S. mutans in the saliva [27].

The antibacterial activities of herbal extracts result from the capacity of their active components to interact with the cell walls of bacteria. Moreover, these antibacterial characteristics are a direct outcome of extracts’ capacity to interact with soluble and extracellular proteins [28].

The sage extract has the greatest total antioxidant, phenolic, and flavonoid levels, according to a phytochemical examination of plant extracts. Phytochemical substances are important elements with antibacterial activity [29]. These are secondary metabolites that plants create as a defensive strategy.

Antioxidant activity is responsible for inhibiting the oxidation of certain substances. It is possible that cell disruption can be brought on by free radicals produced during this oxidation process. Antioxidant chemicals could help remove free radicals that have been produced, facilitating the end of the oxidation process [19]. Alizadeh Behbahani and Imani Fooladi [30] reported similar results, noting that the antioxidant level was related to the overall phenolic content.

Tetrahydroxyflavone, a flavone molecule with many hydroxyl groups and the ability to donate hydrogen radicals was the primary component of the sage extract [31].

Citronellal, a monoterpene mostly generated by the secondary metabolisms of plants, is present in lemongrass extract [32]. Citronellal has various biological effects, including participation in important antibacterial and antioxidant functions.

**Figure 3:**
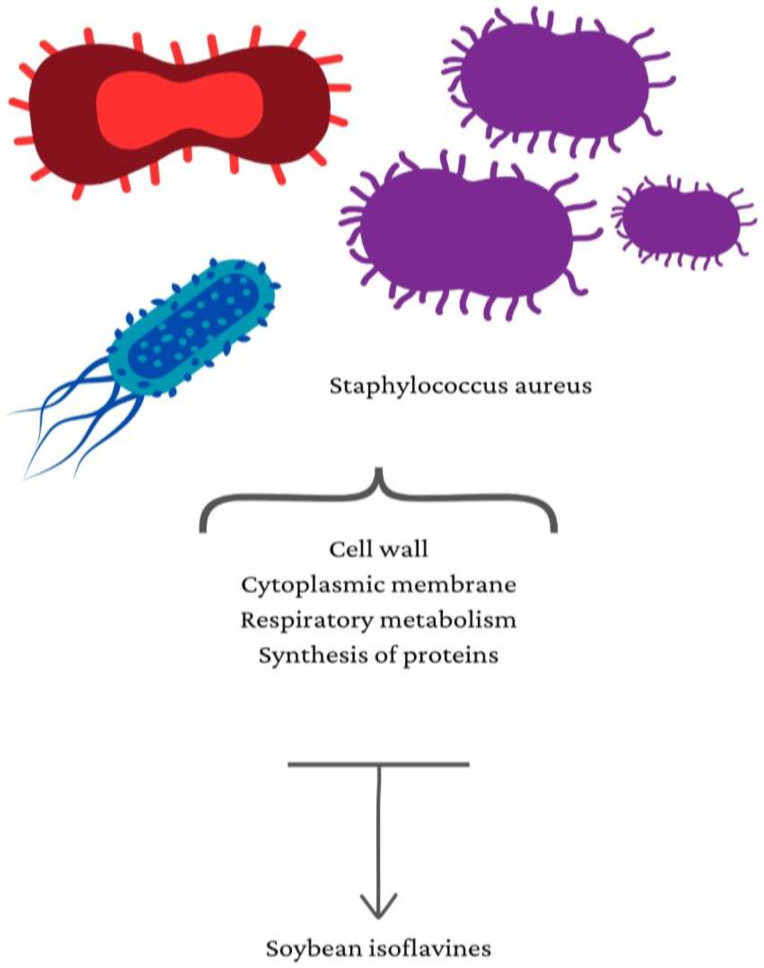
Mechanism of soybeans on *Staphylococcus aureus*.

Moreover, isoflavones from soybeans showed antimicrobial action. Soybean isoflavones inhibited the unwinding of DNA by influencing topoisomerase I and II enzymes, lowering nucleic acid production, increasing supercoiled DNA, and resultantly preventing cell division in bacteria in a study on *Staphylococcus aureus* [33], as shown in Figure 3 below:

The active ingredient in licorice, or Glycyrrhiza glabra, is triterpene saponin glycyrrhizin. Its extracts or fractions have been proven to have a suppressive impact on bacteria such as *S. mutants* in an in vitro investigation [34].

Coffee plants (Coffea arabica and allied species) are high in chlorogenic acid: a phenolic substance with antibacterial action. Chlorogenic acid in green coffee beans was shown to be as effective against *S. mutans* without any known negative effects in a randomized controlled trial [35].

Herbs for this purpose might include a wide variety of plants, such as Aloe vera, Allium sativum, Cinnamomum cassia, Citrus Limon, Eucalyptus globulus, Mentha arvensis, Mentha piperita, Rosmarinus officinalis, and Salvia officinalis [36,37,38,39].

## 4. Herbal Remedies and Periodontal Health

Bacteria are widely accepted as the root cause of periodontal disease. So, it may be beneficial to treat periodontal diseases by eliminating or at least limiting the microorganisms that cause them. Knowing that plaque is directly responsible for 20% of the risk of obtaining periodontitis is crucial [40]. *P. gingivalis*, *B. forsythus*, *A. actinomycetemcomitans*, *T. denticola*, *P. intermedia*, *C. rectus*, *F. nucleatum*, and *C. rectus* are the most well-studied bacteria linked to plaque-induced gingivitis and periodontitis [41].

**Figure 4:**
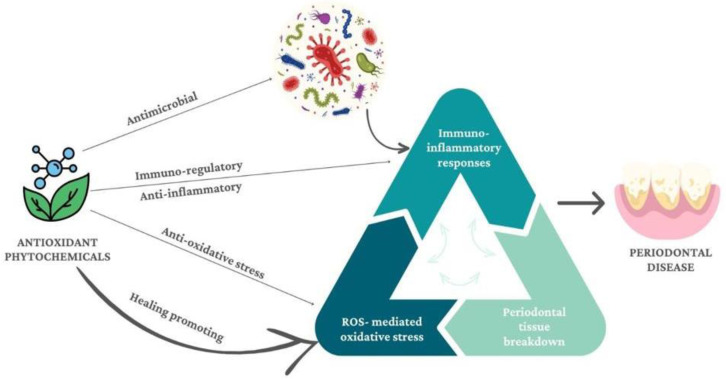
Herbal influence on periodontal disease.

The presence of bacteria triggers immune system activation, which leads to the generation of pro-inflammatory cytokines and, ultimately, reactive oxygen species (ROS). Periodontal structure decays are destroyed because of this chronic inflammatory illness [42], as shown in Figure 4 below:

Several clinical investigations have investigated the use of herbal supplements for the preservation of periodontal health, with mixed results. All the medicines studied in this area demonstrated efficacy in animal studies.

Specifically, tannins found in an extract of the plant Anacardium occidentale have been shown to block the cyclooxygenase (COX) and arachidonic acid (AA) metabolic pathways [43]. When compared to chlorhexidine gluconate (CHX), mouthwash containing this extract has shown similar efficiency in lowering plaque index (PI) and bleeding index (BI) but with fewer adverse effects [44].

Soy isoflavones and other bioflavonoids have been shown to reduce bacterial pathogen populations, slow plaque buildup, and hasten gingival and periodontal repair, all of which contribute to preventing periodontal disorders [45].

Dentifrices and therapeutic chips are only two examples of useful products that herbal medicine has helped bring to market. One study found that *S. persica*-containing dentifrice was more effective thana regular toothpaste at reducing the sulcular bleeding index in patients with gingivitis after three weeks of use. This study was conducted by Azaripour A. et al. [46]. Clinical trials of an herbal dentifrice containing Carica papaya produced encouraging results. After 4 weeks of use, it reduced bleeding on the interdental brushing index just as effectively as a sodium lauryl sulfate-free enzyme-containing dentifrice [47].

The cardiovascular biomarkers C-reactive protein and low-density lipoprotein were reduced, and the high-density lipoprotein level was increased in a 2017 clinical study involving 45 patients with chronic periodontitis who also received 0.2% *C. citratus* (twice daily for three months) [48].

When H. perforatum was tested against isolated samples of oral lactobacillus, it was found that it had antibacterial action, as demonstrated by Nezhad et al. in 2017. Based on their findings, this plant’s hypericin, which had a MIC of 0.625 against acid-producing strains in the mouth, could be used as a replacement for traditional mouthwash and oral disinfectants [49].

Patients receiving fixed orthodontic procedures also experienced the challenge of keeping their teeth clean and free of plaque and tartar. By a wide margin, *S. persica* mouthwash outperformed both chlorhexidine gluconate (CHX) and A. indica extract mouthwashes in reducing the modified bonded bracket plaque index in patients undergoing orthodontic treatment [50].

## 5. Herbal Remedies and Endodontic Treatments

An infected root canal system has the solid residue, fluid streams, and microorganisms necessary to support a microbial community [51]. Endodontic infections are typically caused by bacteria that have spread across the body. With a frequency ranging from 24% to 77%, *Enterococcus faecalis* is one of the most prevalent bacteria in infected root canals and cases of failure root canal treatments requiring retreatment [52]. *E. faecalis* may regulate pH homeostasis, influence host reactions, and consist of digestive enzymes that break down proteins. It has been shown to stick to dentin, invade dentinal tubules, and even survive at Sodium hypochlorite (NaOCl) concentrations as high as 6.5%, which is higher than the amounts typically employed as an antiseptic. Thus, identifying an effective irrigating agent for use against *E. fecalis* is important. The capacity to penetrate the dentinal tubules is crucial. Numerous in vitro investigations have sought out suitable herbal and natural alternatives to intracanal irrigating chemicals; nevertheless, clinical evidence is lacking [53].

Pradhan MS et al. [54] aimed to make an herbal sodium hypochlorite solution by combining 6% sodium hypochlorite with 10% extracts of three herbal species (*C. citarus*, *M. piperita*, and *O. sanctum*). The authors hypothesized that the simultaneous antibacterial effect of this mixture might be achieved without sacrificing pH or chlorine levels. Root canal obturation in deciduous teeth has traditionally been conducted with zinc oxide eugenol; however, this substance has its drawbacks. There have been efforts to find alternatives to eugenol that do not have the same negative effects, such as irritating periapical tissues or bone necrosis [55].

Mechanical preparation alone is inefficient in eliminating pulpal remnants and bacteria from root canals because microorganisms are present in all portions of the root canal system, especially in lateral canals, anastomoses, and dentinal tubules [56]. A variety of chelating compounds, including citric acid, ethylenediaminetetraacetic acid (EDTA), and maleic acid, have been used to remove the smear layer.

Ok, et al., proved that an oregano extract solution had the same effect as NaOCl in removing the smear layer and a strong antibacterial effect [57]. Noni juice, citrus, and carbonic acid juice were shown to have chelating effects better than EDTA when irrigated for 30 min [58]. The inadequate debridement of the root canal system and procedural errors have both been associated with the failure of nonsurgical endodontic treatment. Most obturation materials are made of gutta-percha [59]. A method for the therapeutically effective removal of this material from the root canal has been created via extensive research and development. Essential oils are used to dissolve gutta-percha, remove smear layers, and kill germs at the dental office [60]. Essential oils are a promising alternative to chloroform as a solvent because of their inherent safety.

Safer, more user-friendly, longer-lasting, less expensive, and less tolerant of microbes, these benefits are just a few provided by herbal remedies used in endodontics. In today’s era of evidence-based medicine, all drugs developed for human consumption must be subjected to rigorous in vitro and in vivo testing. Although herbal medicines show promise in vitro, they must first be tested for biocompatibility and safety in both preclinical and clinical studies before they can be recommended for use in endodontics.

## 6. Herbal Remedies and Anticancer Effects

The battle against oral cancer is difficult because of issues including late clinical detection, poor prognosis, and few expensive treatment options. Oral squamous cell carcinoma (OSCC) patients often undergo standard treatments such as radiation, chemotherapy, and surgery. Despite these efforts, the mortality rate from OSCC remains high [61]. Proteolytic enzymes are essential for cancer cell spread because they degrade the extracellular matrix. Matrix metallopeptidase MMP-2 and MMP-9 have the highest associations with neoplastic cell invasion and metastasis [62].

The plant known as Eclipta prostrate may be found in its natural habitat across Asia. By reducing MMP-2 expression, *E. prostrata* extract reduces SCC (squamous cell carcinoma) cell movement and invasion. Reducing MMP-2 expression is a potentially useful method for halting the spread of SCC.

Honeybees collect resinous materials from plants to create propolis: a natural resinous substance. The polyphenolic compounds found in propolis have anticarcinogenic potential, and the most prominent bioactive components of propolis, including CAPE (caffeic acid phenethyl ester), artepillin C, and chrysin, can suppress cancer cell development in a variety of cancer types [63,64].

Piper nigrum (Piperine) was identified in another investigation to have therapeutic potential for regulating inflammatory responses and preventing/attenuating carcinogenesis [65].

**Figure 5:**
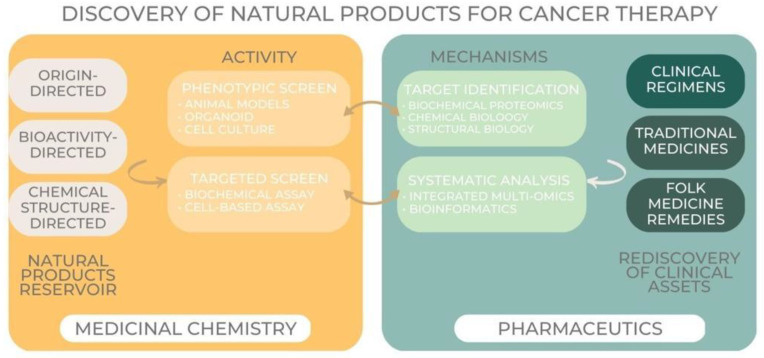
Herbal products mechanism and activity in cancer therapy [66].

Because of their chemical variety, natural products have been studied for their ability to fight cancer for over fifty years. The scientific community as a whole has made great strides, allowing for the clinical use of natural products and the discovery of novel therapeutic prospects, but there is still more work to be done. Given the dramatic shifts in cancer treatment and the increasing importance of cutting-edge technology, it may be time to reevaluate our approaches to learning about and testing the therapeutic potential of compounds found in nature, as mentioned in Figure 5 below:

## 7. Herbal Remedies and Dentifrice

The prevention and management of gingivitis and periodontal disorders rely heavily on effective plaque reduction. There are a variety of mechanical plaque control aids that are available for use today. Plaque may be effectively removed from teeth by brushing them twice a day and using floss once a day. Gingivitis affects an average of three out of every four teeth in adults, yet this condition is still extremely common (affecting more than 50% of the population) [67,68]. To combat plaque and gingivitis, numerous chemical combinations have been tested in dentifrices [69,70].

An increasing number of people are looking for natural alternatives to conventional dentifrices because of concerns over the potentially harmful consequences of the chemicals used in conventional brands. Herbal dentifrices have been the subject of several clinical investigations showing excellent results in reducing plaque and gingivitis above the gum line [71].

Research by Jayashankar et al. [72] looked at the effects of a polyherbal dentifrice that included many different herbs and spices (*Acacia chundra*, *Adhatoda vasica*, *Mimusops elengi*, *Piper nigrum*, *Pongamia pinnata*, *Quercus infectoria*, *Syzygium aromaticum*, *Terminalia chebula*, and *Zingiber officinale*). The results showed a considerable reduction in plaque, gingivitis, and bleeding when compared to the baseline.

Plaque and bleeding indices were also shown to be much lower in the herbal dentifrice group compared to the placebo group, according to research by Willerhausen et al. [73]. Both the control group and the experimental group showed an increase in the alkalinity of their saliva compared to the first reading. Yet, there was absolutely no discernible difference between them.

The contents of the test dentifrice cannot rule out the possibility of hypersensitivity to herbal ingredients. For certain people, cinnamon can trigger an allergic response known as cinnamon contact stomatitis. Cinnamon-flavored chewing gums have been linked to 35 cases, 24 of which were female, according to a recent review [74]. It is important for both patients and doctors to be aware of the possibilities for Cinnamon allergies and the fact that avoiding this spice may be all that is needed for diagnosis and treatment.

In Table 1 below, we summarize the herbal dentifrice and its influence on the oral cavity, as found in the literature research.

## 8. Herbal Products and Antifungal Activities

The use of topical antifungal medicines to treat fungal infections is not without its drawbacks, such as localized burning and redness [84,85]. Because of the quick medication release, drug penetration may be inadequate in certain patients, necessitating continuous therapy. These medications are unlikely to reach the affected area, which leaves the disease untreated. The fact that antifungal resistance is a problem for all presently used antifungal medications [86] makes it a major issue in the development of novel therapeutic approaches to fungal diseases.

There is a proliferation of multidrug-resistant fungal strains calling for both new and improved drug delivery technologies and newer natural antifungal classes. Recent studies on medicinal plants have shown their great pharmacological importance due to the presence of active phytoconstituents.

Natural plant extracts and oils that are used to create antifungal medications might be the answer to this issue. Several plants have been utilized in the development of antimycotic drugs due to their significant antifungal effects [87]. They include cinnamon, peppermint, anise, citronella, pepper, clove, and camphor.

## 9. Research into 3D-Printed Biomaterials and Natural Products for Orthodontics: A Promising Area of Study

Complex 3D biomedical device design and fabrication is a must for the future of healthcare. Technology developments in simulation, data analysis and 3D imaging and rapid prototyping (RP) are also transforming orthodontic clinical workflow [88,89,90,91,92,93]. 

Three-dimensional printing, or 3DP, was developed at MIT (Massachusetts Institute of Technology) and involved inkjet printing a liquid binder solution onto a powder bed to create a three-dimensional object. Since most biomaterials are in either a solid or a liquid form, a large variety of materials have been used in printing. The most common types of this technology used for orthodontics are stereolithography (SLA) and digital light processing (DLP) [94,95]. Modern dental aligners, occlusal splints, surgical splints, indirect bonding trays, and positioning guides for mini-screw insertion are all examples of dental models made using 3D printing technology [96].

The utilization of numerous resins in a single build and an expansion of a library of photocrosslinkable polymers are two of the most recent developments in SLA. More and more aliphatic polyester-containing polymers have been synthesized in recent years because of their ability to biodegrade. The encapsulation of cells during processing and the availability of SLA resins containing biodegradable compounds have both grown recently [96].

Dental models prototyped using both 3D-printing techniques have been shown to be accurate and sufficient for diagnostic and treatment planning purposes, including the creation of dental models for clear aligners, in several prior studies [97].

However, orthodontic treatments should not only have malocclusion correction and aesthetic improvements as their goals. Professionals should instruct patients on preventative dental care, caries management, and new habit formation while they are undergoing orthodontic treatment [98,99]. Oral biofilm undergoes significant alterations during orthodontic treatment because of the increase in retention niches created by orthodontic appliances. 

Chemical and mechanical methods have been employed for controlling dental bacteria biofilms, not only synthetic compounds classically used, or adjuvant therapies such as laser of photodisinfection [100,101,102,103]. The therapeutic advantages of various natural items, herbs, and plant extracts have been investigated in soft tissues as a means of overcoming these unwanted side effects [104], such as melaleuca oil [105], aloe vera [106], honey [107,108] or Matricaria chamomile [109]. Moreover, these herbal extracts can also be applied in the fixed orthodontic appliance of patients undergoing orthodontic treatment to eradicate dental plaques that usually seem to be difficult to remove. However, complementary studies in clinical settings are required on this subject.

## 10. Emerging Trends and Patterns

Several novel pharmaceutical lead chemicals have their origins in the natural products extracted from medicinal plants, which have been shown to be rich sources of physiologically active molecules. Just around one percent of the world’s plant species have been studied phytochemically, so there is a lot of room for new bioactive substances to be found among the other four hundred thousand. Most of these studies have been performed in laboratory or preclinical settings. The efficacy, safety, cost-effectiveness, and characterization of these natural chemicals are all crucial areas that need immediate attention and more financing from the scientific community.

Unfortunately, the low solubility, instability, and bioavailability of these vital compounds, as well as the gastric degradation that happens in the gastrointestinal system, limit their potential therapeutic benefits [110]. New medication delivery methods have been designed as flexible assemblies to overcome the disadvantages of herbal extracts. Enclosing antimicrobial activity in nanoparticles is another way to circumvent resistance mechanisms [111].

## 11. Discussion

Focusing on various aspects of oral pathology, such as tooth decay, endodontic and periodontal disease, oral cancer, and fungal infections, this review article aimed to extract relevant data from the medical literature from a viewpoint of practical applicability and to provide a comprehensive evaluation of the effects and applications of natural biomaterials in dentistry. The next step was to make this mountain of data more digestible and useful for researchers and professionals in a variety of sectors.

The majority of bioactive substances with therapeutic characteristics and pharmaceutical purposes have historically originated from natural sources. In recent years, natural medicinal items have received a lot of attention in the dental care literature alongside commercially manufactured choices. The term “natural bioactive materials” can be used to describe chemical substances derived from plant or animal sources, which have been isolated, purified, and standardized, and which project a specific reaction after establishing a link between their interface or receptors and biological tissues or cells.

Several natural compounds with proven medical efficacy have either already been formulated into standard medicines or are in various stages of development and research. Because of their wide range of biological activity, low cost, and biocompatibility, natural components are often favored over synthetic ones.

There are several potential benefits of herbal treatment even in older patients that are known to be more susceptible to a number of oral and systemic diseases [112]. The synergy between active components in some plants has been demonstrated to have preventative effects, stimulate the regulatory action of the defensive processes of the body, and prepare the body for potential activity against external agents, making certain plants more successful at healing the body than pharmaceuticals [113,114].

Better tolerance and adaptability mean fewer adverse effects and longer-lasting therapeutic results. Herbal medicine, unlike prescription pharmaceuticals, may cure several symptoms at once or be used in conjunction with conventional treatments [115]. The latter requires caution, especially when mixing substances for which there is no clear medical need [116,117].

In order to establish the efficacy of these plants, more study is required, and this groundbreaking effort must be supported and continued.

Adopting a scientific stance towards herbal medicine—one that is critical and skeptical yet open to new information—is crucial. More study is needed to see how useful they might be as medicinal ingredients or therapies. However, there is a risk of overuse or adulteration; thus, caution must be exercised while advocating for herbal medications despite their medicinal potential. It is crucial that the efficacy of herbal medicine be optimized via careful attention to both plant origin and quality control.

To ascertain the safety, appropriate dose, bioavailability, and bioefficacy of herbs and spices, placebo-controlled clinical studies are required. Many plant extracts have anti-inflammatory properties and reduce bleeding, which is helpful in dental treatment; nonetheless, it is of greatest significance to understand the interactions of plant extracts with the body and other drugs. The impact of spices on the body as a whole must be understood, and suitable standards established, which may later require customization based on specific genetic profiles.

## 12. Conclusions and Perspectives

We may conclude that there is a rising interest in the use of herbal items in alternative medicine for the prevention and treatment of dental disease. 

The potential health benefits of herbs and spices are as yet largely unexplored. A lack of uniformity and comparability between different studies may delay the implementation of certain uniform clinical protocols. 

It is clear, however, that a change in the treatment paradigm of oral diseases is necessary. Alternatives such as plant medicines have become an option for treatment and prevention, although there are studies, which do not provide adequate proof of the product’s safety or biocompatibility.

Herbal medicine and other forms of alternative medicine can represent an alternative solution, especially for those living in countries with few resources. That is why it is important that future research use proven and current methods and methodologies.

Herbal medicine is not a passing trend but rather a comprehensive system that incorporates not just plant-based medicinal agents but also homeopathy, acupuncture, and other types of psychotherapy. Plants have been suggested as a viable alternative treatment for oral diseases when efficiency and long-term effects are crucial. It is important for the public good that the next generation of medical experts can take conventional wisdom, modernize it, and incorporate it into the tools of cutting-edge medicine.

Therefore, the use of plants has a long heritage in dentistry, and studies have been ongoing to find further natural solutions to existing problems.

## Figures and Tables

**Table 1:** Herbal dentifrice and their actions.

Mouth Fresheners	Gum Troubles	Tooth Ache	Tooth Cleaning
Mentha [74]	Myrrth [75]	Ginger [76]	Berberis [77]
Rosemary [74]	Neem [78]	Clove [79]	Lemon [80]
Lemon [80]	Ginseng [81]	Turmeric [77]	Guava [77]
Parsley [80]	Green tree [82,83]	Capsicum [79]	Cinnamon [74]

## References

[1] Listl S., Galloway J., Mossey P., Marcenes W. (2015). Global economic impact of dental diseases. J. Dent. Res..

[2] Manipal S., Hussain S., Wadgave U., Duraiswamy P., Ravi K. (2016). The mouthwash war—Chlorhexidine vs. Herbal mouth rinses: A meta-analysis. J. Clin. Diagn. Res..

[3] Haque M.M., Alsareii S.A. (2015). A review of the therapeutic effects of using miswak (*Salvadora persica*) on oral health. Saudi Med. J..

[4] Kumar G., Jalaluddin M., Rout P., Mohanty R., Dileep C.L. (2013). Emerging trends of herbal care in dentistry. J. Clin. Diagn. Res..

[5] Xue W., Yu J., Chen W. (2018). Plants and their bioactive constituents in mesenchymal stem cell-based periodontal regeneration: A novel prospective. BioMed Res. Int..

[6] Chang B., Ahuja N., Ma C., Liu X. (2017). Injectable scaffolds: Preparation and application in dental and craniofacial regeneration. Mater Sci. Eng. R Rep..

[7] Castaneda O.A., Lee S.C., Ho C.T., Huang T.C. (2017). Macrophages in oxidative stress and models to evaluate the antioxidant function of dietary natural compounds. J. Food Drug Anal..

[8] Mao X., Gu C., Chen D., Yu B., He J. (2017). Oxidative stress-induced diseases and tea polyphenols. Oncotarget.

[9] Kim D.H., Suh J., Surh Y.J., Na H.K. (2017). Regulation of the tumor suppressor PTEN by natural anticancer compounds. Ann. N. Y. Acad. Sci..

[10] Siddiqui I.A., Sanna V. (2016). Impact of nanotechnology on the delivery of natural products for cancer prevention and therapy. Mol. Nutr. Food Res..

[11] Siddeeqh S., Parida A., Jose M., Pai V. (2016). Estimation of antimicrobial properties of aqueous and alcoholic extracts of Salvadora persica (miswak) on oral microbial pathogens—An invitro study. J. Clin. Diagn. Res..

[12] Nagpal M., Sood S. (2013). Role of curcumin in systemic and oral health: An overview. J. Nat. Sci. Biol. Med..

[13] Gao L., Xu T., Huang G., Jiang S., Gu Y., Chen F. (2018). Oral microbiomes: More and more importance in oral cavity and whole body. Protein Cell.

[14] Patil S., Rao R.S., Sanketh D.S., Amrutha N. (2013). Microbial flora in oral diseases. J. Contemp. Dent. Pract..

[15] Prabhuswamy B., Mallikarjun N., Nagaraj K., Simpi B. (2018). Comparative evaluation of anticariogenic activity of commercially available herbal dentifrices. SRM J. Res. Dent. Sci..

[16] Khalifa L., Shlezinger M., Beyth S., Houri-Haddad Y., Coppenhagen-Glaze S., Beyth N., Hazan R. (2016). Phage therapy against Enterococcus faecalis in dental root canals. J. Oral. Microbiol..

[17] McCormack M.G., Smith A.J., Akram A.N., Jackson M., Robertson D., Edwards G. (2015). Staphylococcus aureus and the oral cavity: An overlooked source of carriage and infection?. Am. J. Infect. Control..

[18] Aliasghari A., Khorasgani M.R., Khoroushi M. (2017). The Effect of vinegar, rose water and ethanolic extract green tea against oral streptococci, an in vitro study. J. Probiotics Health.

[19] Balakrishnan B., Paramasivam S., Arulkumar A. (2014). Evaluation of the lemongrass plant (*Cymbopogon citratus*) extracted in different solvents for antioxidant and antibacterial activity against human pathogens. Asian Pac. J. Trop. Dis..

[20] Soliman F.M., Fathy M.M., Salama M.M., Saber F.R. (2016). Comparative study of the volatile oil content and antimicrobial activity of *Psidium guajava* L. and *Psidium cattleianum* Sabine leaves. Bull. Fac. Pharm. Cairo Univ..

[21] Fernandes M.R.V., Dias A.L.T., Carvalho R.R., Souza C.R.F., Oliveira W.P. (2014). Antioxidant and antimicrobial activities of *Psidium guajava* L. spray dried extracts. Ind. Crops Prod..

[22] Zulfa Z., Chia C.T., Rukayadi Y. (2016). In vitro antimicrobial activity of *Cymbopogon citratus* (lemongrass) extracts against selected foodborne pathogens. Int. Food Res. J..

[23] Wijesundara N.M., Rupasinghe H.P.V. (2018). Essential oils from Origanum vulgare and Salvia officinalis exhibit antibacterial and anti-biofilm activities against Streptococcus pyogenes. Microb. Pathog..

[24] Zahin M., Ahmad I., Aqil F. (2017). Antioxidant and antimutagenic potential of *Psidium guajava* leaf extracts. Drug Chem. Toxicol..

[25] de Menezes Patrício Santos C.C., Salvadori M.S., Mota V.G., Costa L.M., de Almeida A.A.C., de Oliveira G.A.L., Costa J.P., de Sousa D.P., de Freitas R.M., de Almeida R.N. (2013). Antinociceptive and antioxidant activities of phytol in vivo and in vitro models. Neurosci. J..

[26] Salama M.T., Alsughier Z.A. (2019). Effect of green tea extract mouthwash on salivary Streptococcus mutans counts in a group of preschool children: An in vivo study. Int. J. Clin. Pediatr. Dent..

[27] Abdelmegid F., Al-Agamy M., Alwohaibi A., Ka’abi H., Salama F. (2015). Effect of honey and green tea solutions on Streptococcus mutans. J. Clin. Pediatr. Dent..

[28] Dubey S. (2016). Comparative antimicrobial efficacy of herbal alternatives (*Emblica officinalis*, *Psidium guajava*), MTAD, and 2.5% sodium hypochlorite against Enterococcus faecalis: An in vitro study. J. Oral Biol. Craniofac. Res..

[29] Chakraborty S., Afaq N., Singh N., Majumdar S. (2018). Antimicrobial activity of Cannabis sativa, Thuja orientalis and *Psidium guajava* leaf extracts against methicillin-resistant Staphylococcus aureus. J. Integr. Med..

[30] Alizadeh Behbahani B., Imani Fooladi A.A. (2018). Evaluation of phytochemical analysis and antimicrobial activities Allium essential oil against the growth of some microbial pathogens. Microb. Pathog..

[31] Filipe P., Silva A.M.S., Seixas R.S.G.R., Pinto D.C.G.A., Santos A., Patterson L.K., Silva J.N., Cavaleiro J.A.S., Freitas J.P., Mazière J.-C. (2009). The alkyl chain length of 3-alkyl-3′,4′,5,7-tetrahydroxyflavones modulates effective inhibition of oxidative damage in biological systems: Illustration with LDL, red blood cells and human skin keratinocytes. Biochem. Pharmacol..

[32] Ehsani A., Alizadeh O., Hashemi M., Afshari A., Aminzare M. (2017). Phytochemical, antioxidant and antibacterial properties of *Melissa officinalis* and *Dracocephalum moldavica* essential oils. Vet. Res. Forum..

[33] Wang Q., Wang H., Xie M. (2010). Antibacterial mechanism of soybean isoflavone on Staphylococcus aureus. Arch. Microbiol..

[34] Bhadoria N., Gunwal M.K., Suryawanshi H., Sonarkar S.S. (2019). Antiadherence and antimicrobial property of herbal extracts (*Glycyrrhiza glabra* and *Terminalia chebula*) on *Streptococcus mutans*: An in vitro experimental study. J. Oral Maxillofac. Pathol..

[35] Yadav M., Kaushik M., Roshni R., Reddy P., Mehra N., Jain V., Rana R. (2017). Effect of green coffee bean extract on Streptococcus mutans count: A randomised control trial. J. Clin. Diagn. Res..

[36] Babaeekhou L., Ghane M. (2021). Antimicrobial activity of ginger on cariogenic bacteria: Molecular networking and molecular docking analyses. J. Biomol. Struct. Dyn..

[37] Banavar Ravi S., Nirupad S., Chippagiri P., Pandurangappa R. (2017). Antibacterial effects of natural herbal extracts on Streptococcus mutans: Can they Be potential additives in dentifrices?. Int. J. Dent..

[38] Duarte S., Gregoire S., Singh A.P., Vorsa N., Schaich K., Bowen W.H., Koo H. (2006). Inhibitory effects of cranberry polyphenols on formation and acidogenicity of Streptococcus mutans biofilms. FEMS Microbiol. Lett..

[39] Yoo S., Murata R.M., Duarte S. (2011). Antimicrobial traits of tea- and cranberry-derived polyphenols against *Streptococcus mutans*. Caries Res..

[40] Eke P.I., Dye B.A., Wei L., Slade G.D., Thornton-Evans G.O., Borgnakke W.S., Taylor G.W., Page R.C., Beck J.D., Genco R.J. (2015). Update on prevalence of periodontitis in adults in the United States: NHANES 2009 to 2012. J. Periodontol..

[41] Colombo A.P.V., Tanner A.C.R. (2019). The role of bacterial biofilms in dental caries and periodontal and peri-implant diseases: A historical perspective. J. Dent. Res..

[42] Silva M.F., Leite F.R.M., Ferreira L.B., Pola N.M., Scannapieco F.A., Demarco F.F., Nascimento G.G. (2018). Estimated prevalence of halitosis: A systematic review and meta-regression analysis. Clin. Oral Investig..

[43] Mota M.L., Thomas G., Barbosa Filho J.M. (1985). Anti-inflammatory actions of tannins isolated from the bark of *Anacardium occidentale* L. J. Ethnopharmacol..

[44] Gomes C.E., Cavalcante D.G., Filho J.E., da Costa F.N., da Silva Pereira S.L. (2016). Clinical effect of a mouthwash containing Anacardium occidentale Linn. on plaque and gingivitis control: A randomized controlled trial. Indian J. Dent. Res..

[45] Tanaka K., Sasaki S., Murakami K., Okubo H., Takahashi Y., Miyake Y. (2008). Freshmen in Dietetic Courses Study II Group. Relationship between soy and isoflavone intake and periodontal disease: The Freshmen in Dietetic Courses Study II. BMC Public Health.

[46] Azaripour A., Mahmoodi B., Habibi E., Willershausen I., Schmidtmann I., Willershausen B. (2017). Effectiveness of a miswak extract-containing toothpaste on gingival inflammation: A randomized clinical trial. Int. J. Dent. Hyg..

[47] Saliasi I., Llodra J.C., Bravo M., Tramini P., Dussart C., Viennot S., Carrouel F. (2018). Effect of a toothpaste/mouthwash containing Carica papaya leaf extract on interdental gingival bleeding: A randomized controlled trial. Int. J. Environ. Res. Public Health.

[48] Subha D.S., Pradeep T. (2017). Periodontal therapy with 0.25%Lemongrass oil mouthwash in reducing risk of cardiovascular diseases: A 3-arm prospective parallel experimental study. Ethiop. J. Health Sci..

[49] Nezhad S.K., Zenouz A.T., Aghazadeh M., Kafil H.S. (2017). Strong antimicrobial activity of *Hypericum perforatum* L. against oral isolates of *Lactobacillus* spp. Cell. Mol. Biol..

[50] Niazi F.H., Kamran M.A., Naseem M., AlShahrani I., Fraz T.R., Hosein M. (2018). Antiplaque efficacy of herbal mouthwashes compared to synthetic mouthwashes in patients undergoing orthodontic treatment: A randomised controlled trial. Oral Health Prev. Dent..

[51] Haldal S., Arafath K.M.Y., Subair K., Joseph K. (2016). Biofilms in endodontics. J. Int. Oral Health.

[52] Choudhary E., Indushekar K.R., Saraf B.G., Sheoran N., Sardana D., Shekhar A. (2018). Exploring the role of *Morinda citrifolia* and *Triphala* juice in root canal irrigation: An ex vivo study. J. Conserv. Dent..

[53] Kayaoglu G., Ørstavik D. (2004). Virulence factors of Enterococcus faecalis: Relationship to endodontic disease. Crit. Rev. Oral Biol. Med..

[54] Pradhan M.S., Gunwal M., Shenoi P., Sonarkar S., Bhattacharya S., Badole G. (2018). Evaluation of pH and chlorine content of a novel herbal sodium hypochlorite for root canal disinfection: An experimental in vitro study. Contemp. Clin. Dent..

[55] Sarrami N., Pemberton M., Thornhill M., Theaker E. (2002). Adverse reactions associated with the use of eugenol in dentistry. Br. Dent. J..

[56] Siqueira J.F., Lima K.C., Magalhães F.A., Lopes H.P., de Uzeda M. (1999). Mechanical reduction of the bacterial population in the root canal by three instrumentation techniques. J. Endod..

[57] Ok E., Adanir N., Ozturk T. (2015). Antibacterial and smear layer removal capability of oregano extract solution. Eur. J. Dent..

[58] Chandrasekhar H. (2021). Evaluation of Natural Chelating Agents in Smear Layer Removal. Int. J. Res. Trends Innov..

[59] Wourms D.J., Campbell A.D., Hicks M.L., Pelleu G.B. (1990). Alternative solvents to chloroform for gutta-percha removal. J. Endod..

[60] El-Hawary S.S., Ezzat S.M., Eid G.E., Abd-El Rhman S.K. (2015). Effect of Certain Essential oils on Dissolution of Three Commercial Gutta-percha Brands. J. Essent. Oil Bear. Plants.

[61] Ren Z.H., Hu C.Y., He H.R., Li Y.J., Lyu J. (2020). Global and regional burdens of oral cancer from 1990 to 2017: Results from the global burden of disease study. Cancer Commun..

[62] Lee Y.J., Park B.S., Park H.R., Yu S.B., Kang H.M., Kim I.R. (2017). XIAP inhibitor embelin induces autophagic and apoptotic cell death in human oral squamous cell carcinoma cells. Environ. Toxicol..

[63] Popova M., Giannopoulou E., Skalicka-Woźniak K., Graikou K., Widelski J., Bankova V., Kalofonos H., Sivolapenko G., Gaweł-Bęben K., Antosiewicz B. (2017). Characterization and Biological Evaluation of Propolis from Poland. Molecules.

[64] Kurek-Górecka A., Rzepecka-Stojko A., Górecki M., Stojko J., Sosada M., Swierczek-Zieba G. (2013). Structure and antioxidant activity of polyphenols derived from propolis. Molecules.

[65] Majdalawieh A.F., Carr R.I. (2010). In vitro investigation of the potential immunomodulatory and anti-cancer activities of black pepper (*Piper nigrum*) and cardamom (*Elettaria cardamomum*). J. Med. Food.

[66] Huang M., Lu J.J., Ding J. (2021). Natural Products in Cancer Therapy: Past, Present and Future. Nat. Prod. Bioprospect..

[67] Barnes V.M., Richter R., DeVizio W. (2010). Comparison of the short-term antiplaque/antibacterial efficacy of two commercial dentifrices. J. Clin. Dent..

[68] Paqué P.N., Karygianni L., Kneubuehler J., Fiscalini L., Wiedemeier D.B., Müller M., Attin T., Thurnheer T. (2022). Microbial approaches for the assessment of toothpaste efficacy against oral species: A method comparison. MicrobiologyOpen.

[69] Oliver R.C., Brown L.J., Loe H. (1998). Periodontal diseases in the United States population. J. Periodontol..

[70] Sedghi L.M., Bacino M., Kapila Y.L. (2021). Periodontal Disease: The Good, The Bad, and The Unknown. Front. Cell. Infect. Microbiol..

[71] Hosadurga R., Boloor V.A., Rao S.N., MeghRani N. (2018). Effectiveness of two different herbal toothpaste formulations in the reduction of plaque and gingival inflammation in patients with established gingivitis e a randomized controlled trial. J. Tradit. Complement. Med..

[72] Jayashankar S., Panagoda G.J., Amaratunga E.A.P.D., Perera K., Rajapakse P.S. (2011). A randomised double-blind placebo-controlled study on the effects of a herbal toothpaste on gingival bleeding, oral hygiene and microbial variables. Ceylon. Med. J..

[73] Willershausen B., Gruber I., Hamm G. (1991). The influence of herbal ingredients on the plaque index and bleeding tendency of the gingiva. J. Clin. Dent..

[74] Calapai G., Miroddi M., Mannucci C., Minciullo P., Gangemi S. (2014). Oral adverse reactions due to cinnamon-flavoured chewing gums consumption. Oral Dis..

[75] Eid R.A.A. (2021). Efficacy of *Commiphora* myrrh mouthwash on early wound healing after tooth extraction: A randomized controlled trial. Saudi Dent. J..

[76] Badooei F., Imani E., Hosseini-Teshnizi S., Banar M., Memarzade M.R. (2021). Comparison of the effect of ginger and aloe vera mouthwashes on xerostomia in patients with type 2 diabetes: A clinical trial, triple-blind. Med. Oral Patol. Oral Y Cir. Bucal.

[77] Vijapur P.V., Vijapur L.S., Yaragattimath P., Raibagi G., Mallapur Y., Desai B. (2022). Formulation and evaluation of herbal mouthwash containing natural ingredients for anti-microbial activity. Int. J. Creat. Res. Thoughts.

[78] Heyman L., Houri-Haddad Y., Heyman S.N., Ginsburg I., Gleitman Y., Feuerstein O. (2017). Combined antioxidant effects of Neem extract, bacteria, red blood cells and Lysozyme: Possible relation to periodontal disease. BMC Complement. Altern. Med..

[79] Shankar S., Gopinath P., Roja E. (2022). Role of Spices and Herbs in Controlling Dental Problems. Res. J. Pharmacol. Pharmacodyn..

[80] Wu X., Zhang J., Zhou Y., He Z., Cai Q., Nie M. (2018). Whether Chinese Medicine Have Effect on Halitosis: A Systematic Review and Meta-Analysis. Evid. Based Complement. Altern. Med..

[81] Patel S., Rauf A. (2017). Adaptogenic herb ginseng (Panax) as medical food: Status quo and future prospects. Biomed. Pharmacother..

[82] Forouzanfar A. (2011). Review of the therapeutic effects of Camellia sinensis (green tea) on oral and periodontal health. J. Med. Plant Res..

[83] Teixeira A.M., Sousa C. (2021). A Review on the Biological Activity of *Camellia* Species. Molecules..

[84] Li M.Y., Wang J., Xu Z.T. (2010). Effect of a variety of Chinese herbs and an herb-containing dentifrice on volatile sulfur compounds associated with halitosis: An in vitro analysis. Curr. Ther. Res. Clin. Exp..

[85] Huang N., Li J., Qiao X., Wu Y., Liu Y., Wu C., Li L. (2022). Efficacy of probiotics in the management of halitosis: A systematic review and meta-analysis. BMJ Open.

[86] Abd Rashed A., Rathi D.G., Ahmad Nasir N.A.H., Abd Rahman A.Z. (2021). Antifungal Properties of Essential Oils and Their Compounds for Application in Skin Fungal Infections: Conventional and Nonconventional Approaches. Molecules.

[87] Fairlamb A.H., Gow N.A., Matthews K.R., Waters A.P. (2016). Drug resistance in eukaryotic microorganisms. Nat. Microbiol..

[88] Lo Giudice A., Ronsivalle V., Rustico L., Aboulazm K., Isola G., Palazzo G. (2022). Evaluation of the accuracy of orthodontic models prototyped with entry-level LCD-based 3D printers: A study using surface-based superimposition and deviation analysis. Clin. Oral Investig..

[89] Sufaru I.G., Macovei G., Stoleriu S., Martu M.A., Luchian I., Kappenberg-Nitescu D.C., Solomon S.M. (2022). 3D Printed and Bioprinted Membranes and Scaffolds for the Periodontal Tissue Regeneration: A Narrative Review. Membranes.

[90] Tatarciuc M., Maftei G.A., Vitalariu A., Luchian I., Martu I., Diaconu-Popa D. (2021). Inlay-Retained Dental Bridges—A Finite Element Analysis. Appl. Sci..

[91] Tărăboanță I., Stoleriu S., Nica I., Georgescu A., Gamen A.G., Maftei G.A., Andrian S. (2020). Roughness variation of a nannohbrid composite resin submitted to acid and abrasive challenges. Int. J. Med. Dent..

[92] Gelețu G.L., Burlacu A., Murariu A., Andrian S., Golovcencu L., Baciu E.-R., Maftei G., Onica N. (2022). Customized 3D-Printed Titanium Mesh Developed for an Aesthetic Zone to Regenerate a Complex Bone Defect Resulting after a Deficient Odontectomy: A Case Report. Medicina.

[93] Venezia P., Ronsivalle V., Rustico L., Barbato E., Leonardi R., Lo Giudice A. (2022). Accuracy of orthodontic models prototyped for clear aligners therapy: A 3D imaging analysis comparing different market segments 3D printing protocols. J. Dent..

[94] Favero C.S., English J.D., Cozad B.E., Wirthlin J.O., Short M.M., Kasper F.K. (2017). Effect of print layer height and printer type on the accuracy of 3-dimensional printed orthodontic models. Am. J. Orthod. Dentofac. Orthop..

[95] Zhang Z.C., Li P.L., Chu F.T., Shen G. (2019). Influence of the three-dimensional printing technique and printing layer thickness on model accuracy. J. Orofac. Orthop..

[96] Kang H.W., Cho D.W. (2012). Development of an Indirect Stereolithography Technology for Scaffold Fabrication with a wide range of biomaterial selectivity. Tissue Eng. Part C Methods.

[97] Hazeveld A., Huddleston Slater J.J., Ren Y. (2014). Accuracy and reproducibility of dental replica models reconstructed by different rapid prototyping techniques. Am. J. Orthod. Dentofacl. Orthop..

[98] Atack N.E., Sandy J.R., Addy M. (1996). Periodontal and microbiological changes associated with the placement of orthodontic appliances. A review. J. Periodontol..

[99] Kado I., Hisatsune J., Tsuruda K., Tanimoto K., Sugai M. (2020). The impact of fixed orthodontic appliances on oral microbiome dynamics in Japanese patients. Sci. Rep..

[100] Luchian I., Goriuc A., Martu M.A., Covasa M. (2021). Clindamycin as an Alternative Option in Optimizing Periodontal Therapy. Antibiotics.

[101] Martu M.A., Rezus E., Popa C., Solomon S.M., Luchian I., Pendefunda A.C., Sioustis I., Anton D., Martu S., Foia L. (2018). Correlations between systemic therapy with conventional (synthetic) and biological DMARDS, rheumatoid arthritis and periodontal indices of chronic periodontitis. Rom. J. Oral Rehabil..

[102] Martu M.-A., Surlin P., Lazar L., Maftei G.A., Luchian I., Gheorghe D.-N., Rezus E., Toma V., Foia L.-G. (2021). Evaluation of Oxidative Stress before and after Using Laser and Photoactivation Therapy as Adjuvant of Non-Surgical Periodontal Treatment in Patients with Rheumatoid Arthritis. Antioxidants.

[103] Martu M.A., Maftei G.A., Luchian I., Stefanescu O.M., Scutariu M.M., Solomon S.M. (2021). The Effect of Acknowledged and Novel Anti-Rheumatic Therapies on Periodontal Tissues—A Narrative Review. Pharmaceuticals.

[104] Rasooli I., Shayegh S., Taghizadeh M., Astaneh S.D. (2008). Phytotherapeutic prevention of dental biofilm formation. Phytother. Res..

[105] Carson C.F., Hammer K.A., Riley T.V. (2006). Melaleuca alternifolia (tea tree) oil: A review of antimicrobial and other medicinal properties. Clin. Microbiol. Rev..

[106] Yeturu S.K., Acharya S., Urala A.S., Pentapati K.C. (2016). Effect of Aloe vera, chlorine dioxide, and chlorhexidine mouth rinses on plaque and gingivitis: A randomized controlled trial. J. Oral Biol. Craniofac. Res..

[107] Atwa A.D., AbuShahba R.Y., Mostafa M., Hashem M.I. (2014). Effect of honey in preventing gingivitis and dental caries in patients undergoing orthodontic treatment. Saudi Dent. J..

[108] Deglovic J., Majtanova N., Majtan J. (2022). Antibacterial and Antibiofilm Effect of Honey in the Prevention of Dental Caries: A Recent Perspective. Foods.

[109] Goes P., Dutra C.S., Lisboa M.R., Gondim D.V., Leitão R., Brito G.A., Rego R.O. (2016). Clinical efficacy of a 1% Matricaria chamomile L. mouthwash and 0.12% chlorhexidine for gingivitis control in patients undergoing orthodontic treatment with fixed appliances. J. Oral Sci..

[110] Mutlu-Ingok A., Devecioglu D., Dikmetas D.N., Karbancioglu-Guler F., Capanoglu E. (2020). Antibacterial, Antifungal, Antimycotoxigenic, and Antioxidant Activities of Essential Oils: An Updated Review. Molecules.

[111] Laffleur F., Keckeis V. (2020). Advances in drug delivery systems: Work in progress still needed?. Int. J. Pharm. X.

[112] Popa C., Filioreanu A.M., Stelea C., Maftei G.A., Popescu E. (2018). Prevalence of oral lesions modulated by patient’s age: The young versus the elderly. Rom. J. Oral Rehabil..

[113] Cañigera S., Dellacasa T., Blandoni A. (2003). Medicinal plants and phytotherapy: Indicators of dependence or developmental factors?. Lat. Am. J. Pharm..

[114] Amiri M.S., Yazdi M.E.T., Rahnama M. (2021). Medicinal plants and phytotherapy in Iran: Glorious history, current status and future prospects. Plant Sci. Today.

[115] Cortez G., Macedo-Ceja J., Hernández-Arroyo M., Arteaga-Aureoles G., Espinosa-Galván D., Rodríguez-Landa J. (2004). Pharmacognosy: Brief history of its origins and their relation with the medical sciences. Rev. Biomed..

[116] Heinrich M. (2000). Ethnobotany and its role in drug development. Phytother. Res..

[117] Cahlíková L., Šafratová M., Hošťálková A., Chlebek J., Hulcová D., Breiterová K., Opletal L. (2020). Pharmacognosy and Its Role in the System of Profile Disciplines in Pharmacy. Nat. Prod. Commun..

